# The Efficacy and Tolerability of ‘Polypills’: Meta-Analysis of Randomised Controlled Trials

**DOI:** 10.1371/journal.pone.0052145

**Published:** 2012-12-19

**Authors:** C Raina Elley, Ajay K. Gupta, Ruth Webster, Vanessa Selak, Min Jun, Anushka Patel, Anthony Rodgers, Simon Thom

**Affiliations:** 1 School of Population Health, University of Auckland, Auckland, New Zealand; 2 International Centre for Circulatory Health, Imperial College, London, United Kingdom; 3 The George Institute for Global Health, University of Sydney, Sydney, Australia; University of British Columbia, Canada

## Abstract

**Background:**

To assess the blood pressure and lipid-lowering efficacy and tolerability of ‘polypills’ used in cardiovascular disease prevention trials.

**Methodology/Principal Findings:**

Systematic review and meta-analysis. *Search strategy:* The Cochrane Central Register of Controlled Trials, Medline, and PubMed databases were searched for eligible trials. *Study inclusion criteria*: Randomised controlled trials of at least six weeks duration, which compared a ‘polypill’ (that included at least one anti-hypertensive and one lipid-lowering medication) with a placebo (or one active component). *Outcome measures:* Change from baseline in systolic and diastolic blood pressures, and total and LDL-cholesterol; discontinuation of study medication and reported adverse effects. Of 44 potentially eligible studies, six trials (including 2,218 patients without previous cardiovascular disease) fulfilled the inclusion criteria. Compared with placebo, ‘polypills’ reduced systolic blood pressure by −9.2 mmHg (95% confidence interval (CI): −13.4, −5.0) diastolic blood pressure by −5.0 mmHg (95%CI: −7.4, −2.6), total cholesterol by −1.22 mmol/L (95%CI: −1.60, −0.84) and LDL-cholesterol by −1.02 mmol/L (95%CI: −1.37, −0.67). However, those taking a ‘polypill’ (vs. placebo or component) were more likely to discontinue medication (20% vs 14%) (Odds ratio: 1.5 (95% CI: 1.2, 1.9)). There was no significant difference in reported adverse effects amongst those on a ‘polypill’ (36% vs. 28%) (OR: 1.3 (95%CI: 0.7, 2.5)). There was high statistical heterogeneity in comparisons for blood pressure and lipid-lowering but use of random-effects and quality-effects models produced very similar results.

**Conclusions/Significance:**

Compared with placebo, the ‘polypills’ reduced blood pressure and lipids. Tolerability was lower amongst those on ‘polypills’ than those on placebo or one component, but differences were moderate. Effectiveness trials are needed to help clarify the status of ‘polypills’ in primary care and prevention strategies.

## Introduction

Cardiovascular disease is the leading cause of death worldwide. [1] On the basis of a substantial body of evidence, cardiovascular guidelines have recommended that those with a past history of cardiovascular disease [2] or who otherwise have a high risk of disease [3] follow lifestyle interventions and receive blood pressure lowering [4] and lipid-lowering medications, [5], [6] and where benefit outweighs risk, aspirin therapy. [7], [8] This combination of therapies substantially reduces risk of future cardiovascular events. [9], [10], [11] Despite guidelines, high proportions of those at high cardiovascular risk are not prescribed these preventive medications, particularly in low income countries. [12], [13], [14] Besides relatively low rates of prescribing of recommended medications, long-term adherence to medications is also low, which further compromises the preventive potential of these medications. A 2003 World Health Organisation (WHO) report estimated that less than 50% of those prescribed long-term medications for chronic conditions take their medications regularly. [15] Each additional cardiovascular medication prescribed tends to be associated with lower adherence. [16] Adherence also reduces sharply in the first year after commencing medication, although adherence is better if medications are initiated together. [15], [17] The WHO report recommends that interventions to improve adherence should be developed and could improve health outcomes to a greater extent than developing new medications. [15] Using fixed dose combinations, or ‘polypills’, that combine generic versions of different classes of preventive medications for high risk individuals is one such strategy, as it may simplify the medication regime for both prescriber and patient and reduce cost for health funder and patient. [18], [19].

In 2001, a WHO and Wellcome Trust meeting of experts concluded that a fixed-dose combination pill containing aspirin, statin and two blood pressure (BP) lowering agents may improve adherence to treatment as well as substantially reduce the cost of the drugs, particularly for low and middle income countries. [20] In 2003, Wald and Law claimed that ischemic heart disease could be reduced by 88% and strokes by 80% if all those over 55 years of age were given a ‘polypill’ containing three low-dose blood pressure lowering medications, a statin, low dose aspirin and folic acid. [11] This controversial approach of ‘medicalising’ the population has been followed by more targeted approaches of ‘polypills’ recommended for high risk individuals only, where effectiveness and cost-effectiveness are likely to be most favourable. [21] An important aspect of the ‘polypills’ is their affordability, particularly for low-income countries where cardiovascular mortality is increasing. [22].

Evidence for fixed-dose combination (FDC) medications has been promising, as shown by a meta-analysis of antihypertensive FDCs. [23] In 2002, the WHO recommended that bioavailability, pharmacokinetics, effects on risk factors and side effects of ‘polypill’ formulations should be assessed by short-term efficacy trials, followed by community-based effectiveness trials and cost-effectiveness evaluations comparing ‘polypills’ to standard practice. [20] It has taken more than 10 years to progress these aims. Several efficacy trials of ‘polypills’ including at least one antihypertensive and one lipid-lowering medication have been conducted. Some are placebo-controlled while others have active component comparators. This systematic review and meta-analysis aimed to assess the efficacy and tolerability of the ‘polypill’ approach by examining the effects on blood pressure, lipid profiles and discontinuation and side effects of medication.

## Methods

### Ethics Statement

This was a meta-analysis of published summary data and therefore did not require ethics approval.

### Definition of a ‘Polypill’

For the purposes of this review, a ‘polypill’ has been defined as a medication formulation containing at least one blood pressure lowering medication and one lipid-lowering medication (with or without an anti-platelet agent such as aspirin).

### Selection of Studies

This meta-analysis included randomised controlled trials of cardiovascular ‘polypills’ that were published in English. Trials of at least six weeks duration were eligible to allow reasonable estimation of clinical effect and likely discontinuation of medication. Trials must have assessed at least one primary outcome of this review, which included systolic and diastolic blood pressure, serum total and LDL-cholesterol and a measure of tolerability, either discontinuation of medication or proportion reporting side effects. The comparator could be placebo or component medications that allowed a placebo comparison for at least one primary outcome.

The Cochrane Central Register of Controlled Trials, Medline, and PubMed databases were searched for eligible trials using the terms in Table S1. The strategy was guided by the Cochrane Systematic Review Handbook. [24] Reference lists were also searched. A second researcher undertook an independent literature search of Medline, PubMed and Embase (Table S2).

### Study Procedures

Data were extracted on design, intervention, duration of follow-up, sample size (intervention and control) and follow-up rate. Study population demographic, cardiovascular risk and co-morbidity characteristics were also recorded. Data extraction was undertaken separately by two researchers. Study quality was assessed using the Jadad criteria where a score out of five is given for description and appropriateness of randomisation and blinding, and for description of withdrawals and drop-outs. [25] The Cochrane criteria for risk of bias were also used to assess study quality. [24] Change in outcome measures in each group over the trial was recorded. Authors were contacted where data were missing. The number and proportion of study participants who discontinued the study medication during the trial and the proportion of participants with side effects were compared between intervention and control groups.

### Statistical Analysis

The weighted mean difference in continuous outcomes was calculated using Cochrane RevMan 5.1 software [24] and checked by a separate researcher using STATA (v12, StataCorp LP). Means and standard deviations of change of the primary outcome measures were used where reported. Where standard deviations could not be obtained from the published data or from contacting the authors, standard deviations from baseline were used. [26] Where there was no placebo control, comparators not containing an anti-hypertensive for blood pressure analyses or not containing a lipid-lowering medication for lipid analyses were used. Odds ratios and 95% confidence intervals were calculated for dichotomous variables. Heterogeneity was investigated using Tau^2^ and I^2^ statistics. Where substantial statistical heterogeneity was found, random-effects models were used and compared with quality-effects models. [27] Publication bias was investigated using Begg’s rank correlation and Egger’s regression methods in STATA v12, and funnel plots in RevMan. [28], [29], [30] Sensitivity analyses were carried out on the basis of duration of follow-up, as it was hypothesized that effect size may reduce if adherence decreased over time.

## Results

**Figure 1:**
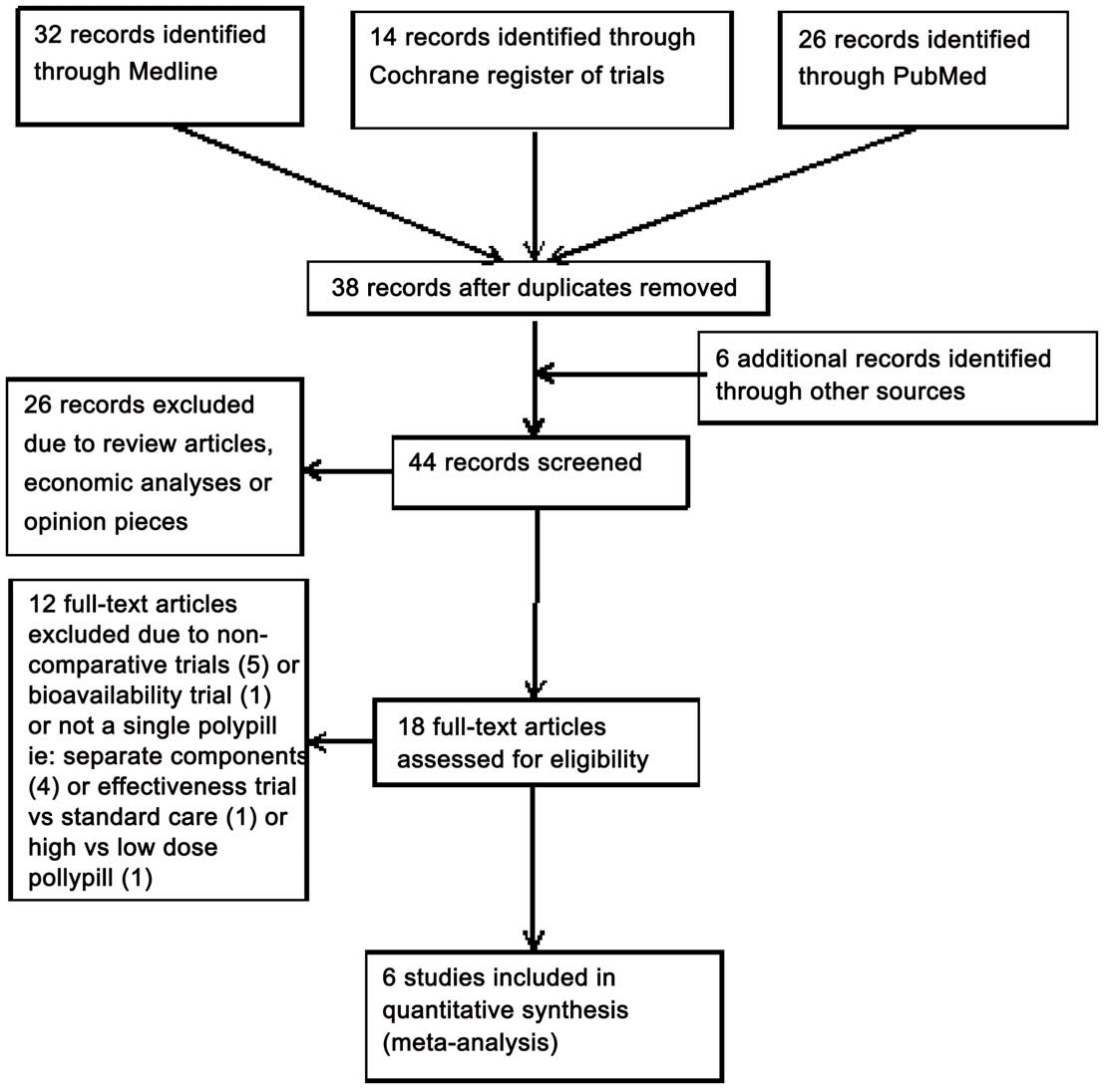
Polypills Meta-analysis ‘PRISMA’ Flow Diagram.

Of the 44 studies identified by the literature search, six fulfilled the inclusion criteria and were included in the meta-analysis (Figure 1 and Table S1). A search undertaken independently by a second researcher did not identify any additional eligible studies (Figure S1). The characteristics and quality of the eligible studies are included in Table 1.

**Table 1:** Baseline Characteristics and Study Quality of included Randomised Controlled Trials.

IncludedStudy	Study population characteristics	Mean age (SD); female gender (%)	Mean SBP(SD)/DBP (SD) mmHg	Mean total cholesterol (SD); LDL (SD) mmol/L	‘Polypill’ contents (dose); n	Comparison; n	Duration of follow-up;	Outcomes assessed;	Study quality (Jadad score [25])
Grimm et al.2010 [31] *	Primary prevention (no previousCVD) Inclusion criteria: Any CVDrisk factor but no diabetes	56 (range 24–84); 50%	132.6 (11.8)/81.5 (8.9)	5.48 (0.78); 3.35 (0.60)	Amlodipine (5–10 mg) Atorvastatin (20 mg); n = 122	Amlodipine (5–10 mg); n = 122	6 weeks	SBP, DBP*, Total cholesterol, LDL, AEs;	Jadad 5/5; 89% follow-up
Malekzadeh et al.2010 [34] *	Primary prevention (no previousCVD) Inclusion criteria: >50/55 yrs,no previous CVD; not on active BPor lipid lowering medications. No exclusionfor diabetes	59.1 (6.9); 33%	127.5 (17.3)/79.8 (10.1)	5.26 (1.01); 2.99 (0.68)	Aspirin (81 mg), Enalapril (2.5 mg), Atorvastatin (20 mg) and Hydrochloro-thiazide (12.5 mg); n = 241	Placebo; n = 234	12 months	SBP, DBP, Total cholesterol, LDL, AEs;	Jadad 4/5; Imbalance in baseline chchs suggests inadequacy of randomisation; Low follow-up rate: 68% in intervention, 78% in control
Neutel et al.2009 [32] *	Primary prevention (no previousCVD) Inclusion criteria: Hypertensionor dyslipidaemia but no diabetesand not on any treatment	52.9 (10.7);54%	146.5 (10.0)/91.1 (6.8)	5.65 (0.72); 3.46 (0.60)	Amlodipine (5 mg) Atorvastatin (20 mg)(plus TLC);n = 66	Placebo (plus TLC); n = 64	8 weeks	SBP, DBP. Total cholesterol, LDL, AEs;	Jadad 4/5; 90% follow-up
Pill Collaborative2011 [33]	Primary prevention (no previousCVD) Inclusion criteria: 5-yr CVD risk>7.5% (based on Framinghamrisk score) or 5%–7.5% and 2 CVDrisk factors. No exclusionfor diabetes	61.4 (7.2); 19%	134.0 (13.5)/80.5 (9.0)	5.50 (1.05); 3.65 (0.90)	Aspirin (75 mg), Lisinopril (10 mg) Hydrochlorothiazide (12.5 mg) and Simvastatin (20 mg); n = 189	Placebo; n = 189	12 weeks	SBP, DBP, Total cholesterol, LDL, AEs;	Jadad 5/5; 99% follow-up and some imbalance in baseline SBP
Wald2012 [36]	Primary prevention (no previousCVD) Inclusion criteria: over50 years of age	59 (range 51–77); 26%	143.0 (16)/86.0 (10)**	5.9 (1.0); 3.7 (0.9)**	Amlodipine (2.5 mg) Losartan (25 mg), Hydrochlorothiazide (12.5 mg) and Simvastatin (40 mg); n = 86	Placebo; n = 86	12 weeks (cross-over RCT)	SBP, DBP, Total cholesterol, LDL, AEs	Jadad 5/5; 98% follow-up
The IndianPolycap Study ‘TIPS’2009 [35] #	Primary prevention (no previous CVD) Inclusion criteria: at least one CV risk factor (including diabetes)	53.6 (7.7); 44%	134.3 (12.3)/85.2 (8.1)	4.7 (0.9); 3.0 (0.8)	Hydrochlorothiazide (12·5 mg), Atenolol (50 mg), Ramipril (5 mg), Simvastatin (20 mg), Aspirin (100 mg); n = 412	Aspirin (100 mg); n = 205 (Simvastatin 20 mg group added for BP comparison n = 202)	12 weeks (some 8–12 weeks);	SBP, DBP, Total cholesterol, LDL, AEs;	Jadad 5/5; 85% follow-up in these three arms

* BP not assessed in meta-analysis as both arms contained an anti-hypertensive;

** Following placebo 12 weeks of cross-over RCT;

# Double-blind 9-arm with varying medication components and number of components. Only three arms were used in this meta-analysis: the polycap, aspirin and simvastatin arms;

BP = blood pressure and measured in mmHg; SBP = systolic blood pressure; DBP = Diastolic blood pressure; Total chol. = total cholesterol in mmol/L; LDL = LDL cholesterol in mmol/L; AE = adverse events; TLC = therapeutic lifestyle changes; SD = standard deviation; CVD = cardiovascular disease.

### Characteristics of Studies

The intervention of all trials was a fixed dose combination that contained either one, two or three antihypertensives (including a calcium channel blocker [31], [32], a thiazide and ACE inhibitor [33], [34], a thiazide, ACE inhibitor and beta-blocker [35], or a thiazide, angiotensin receptor blocker and a calcium channel blocker [36]) plus one lipid lowering medication (including atorvastatin (20 mg) [31], [32], [34] or simvastatin (20 mg [33], [35] or 40 mg [36])). Three ‘polypills’ also included aspirin (75 mg or 100 mg). [33], [34], [35] The comparison was either a true placebo [32], [33], [34] or one cardiovascular component (aspirin [35], simvastatin [35] or amlodipine [31]). All trials were double-blind. Five were parallel designs and one was a cross-over design. [36] One parallel trial included nine arms of varying numbers of fixed dose components but with no placebo arm. [35] For this trial only the ‘polycap’ arm and the arms not containing an antihypertensive (‘aspirin arm’ and ‘statin arm’) for blood pressure comparisons and one arm not containing a lipid-lowering agent (‘aspirin arm’) for lipid comparisons were used. [35] Five studies were of 6–12 weeks and one trial of 12 months duration. [34] Two other randomised controlled trials of ‘polypills’ were identified but excluded. One study compared low-dose with high-dose polycap components but did not include a placebo arm or reduced number of components that allowed a placebo comparison of blood pressure or serum lipid concentrations. [37] The other study was an open label trial comparing a ‘polypill’ with usual care. [38].

### Participant Characteristics

Table 1 summarises the study and participant characteristics. A total of 2,218 patients were included in the meta-analysis comparisons. This was made up of 1,116 in a ‘polypill’ group and 1,102 in a comparison group. No participants had previous cardiovascular disease but most had at least one cardiovascular risk factor. Despite this, there were some differences between study populations. Two trials excluded those with diabetes. [31], [32] Mean baseline systolic blood pressure varied from 125 mmHg [34] to 147 mmHg [32] in the intervention groups across trials and the proportion of women participants varied from 19% [33] to 53%. [31] All trials except two [35], [36] allowed concomitant blood-pressure lowering medication, although levels of use were low.

### Effect on Blood Pressure

**Figure 2:**
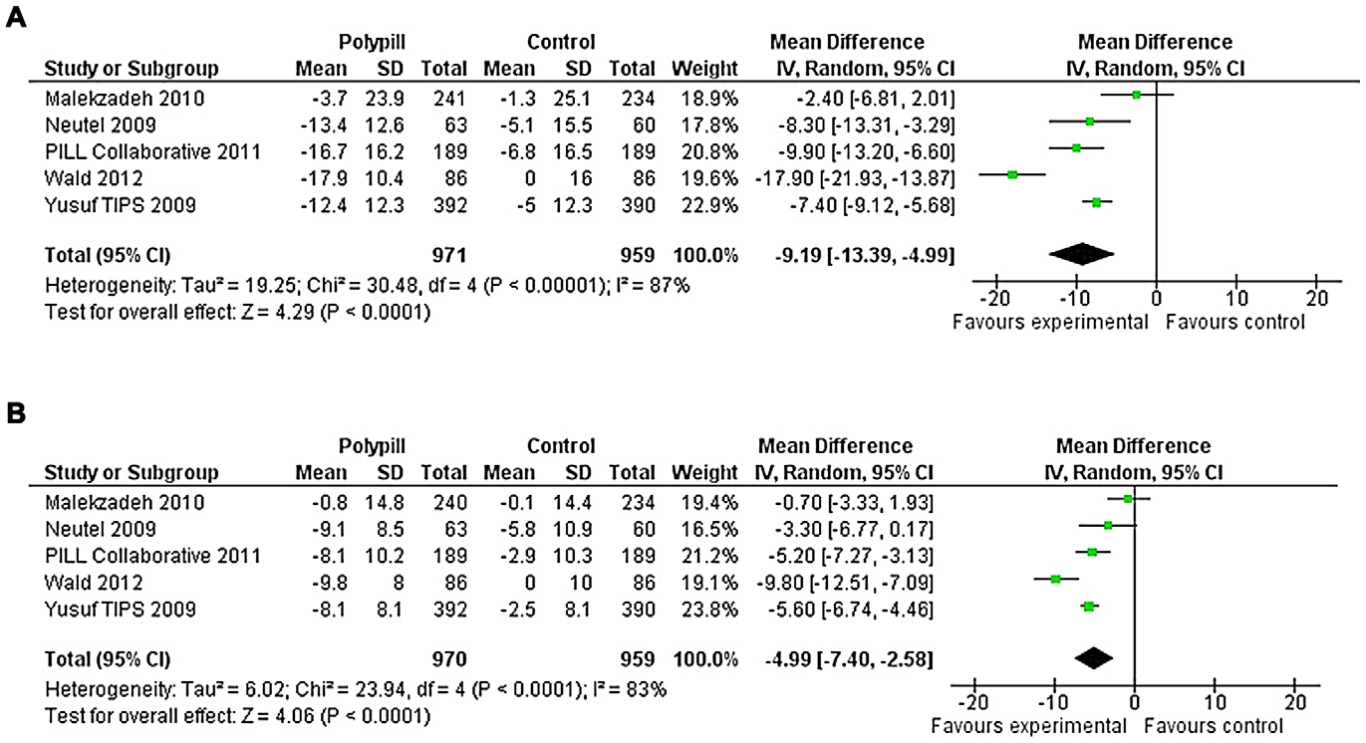
Forest Plots of Polypills versus Control for Change in Systolic and Diastolic Blood Pressure.

Results from five trials, where control arms did not contain antihypertensive medication, were combined to assess effects of ‘polypills’ on blood pressure lowering (Figure 2). Compared with placebo, the ‘polypills’ reduced systolic blood pressure by −9.2 mmHg (95% confidence interval (CI): −13.4, −5.0) and diastolic blood pressure by −5.0 mmHg (95%CI: −7.4, −2.6). However, there was evidence of significant heterogeneity of trials (I^2^ 87% and 83%, respectively). A sensitivity analysis including the four shorter trials of 6 to 12 weeks duration and excluding the longer trial of 12 months [34], found systolic blood pressure reduced by −10.8 mmHg (95%CI: −15.2, −6.3), and diastolic blood pressure by −6.0 mmHg (95%CI: −8.1, −4.0).

### Effect on Lipid Profiles

**Figure 3:**
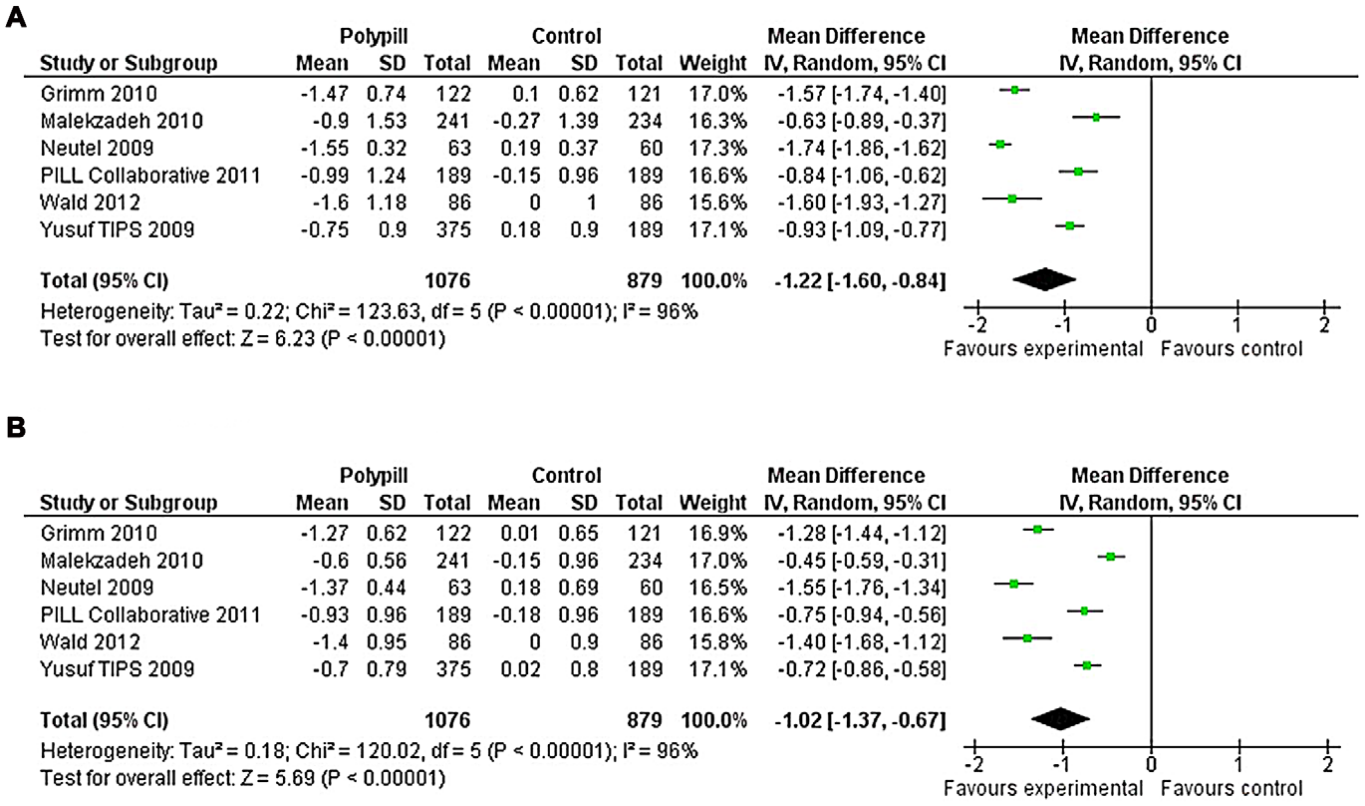
Forest Plots of Polypills versus Control for Change in Total Cholesterol and LDL-cholesterol.

Results from all trials, where the control did not contain lipid-lowering medication, were combined to assess effects on serum lipids (Figure 3). Compared with placebo, the ‘polypills’ reduced total cholesterol by −1.22 mmol/L (95%CI: −1.60, −0.84) and LDL-cholesterol by −1.02 mmol/L (95%CI: −1.37, −0.67). There was high statistical heterogeneity (I^2^ = 96%). If the 12 month trial was excluded [34], total cholesterol reduced by −1.33 mmol/L (95%CI: −1.72, −0.95) and LDL-cholesterol by −1.13 mmol/L (95%CI: −1.47, −0.79).

### Discontinuation of Study Medication and Side Effects

**Figure 4:**
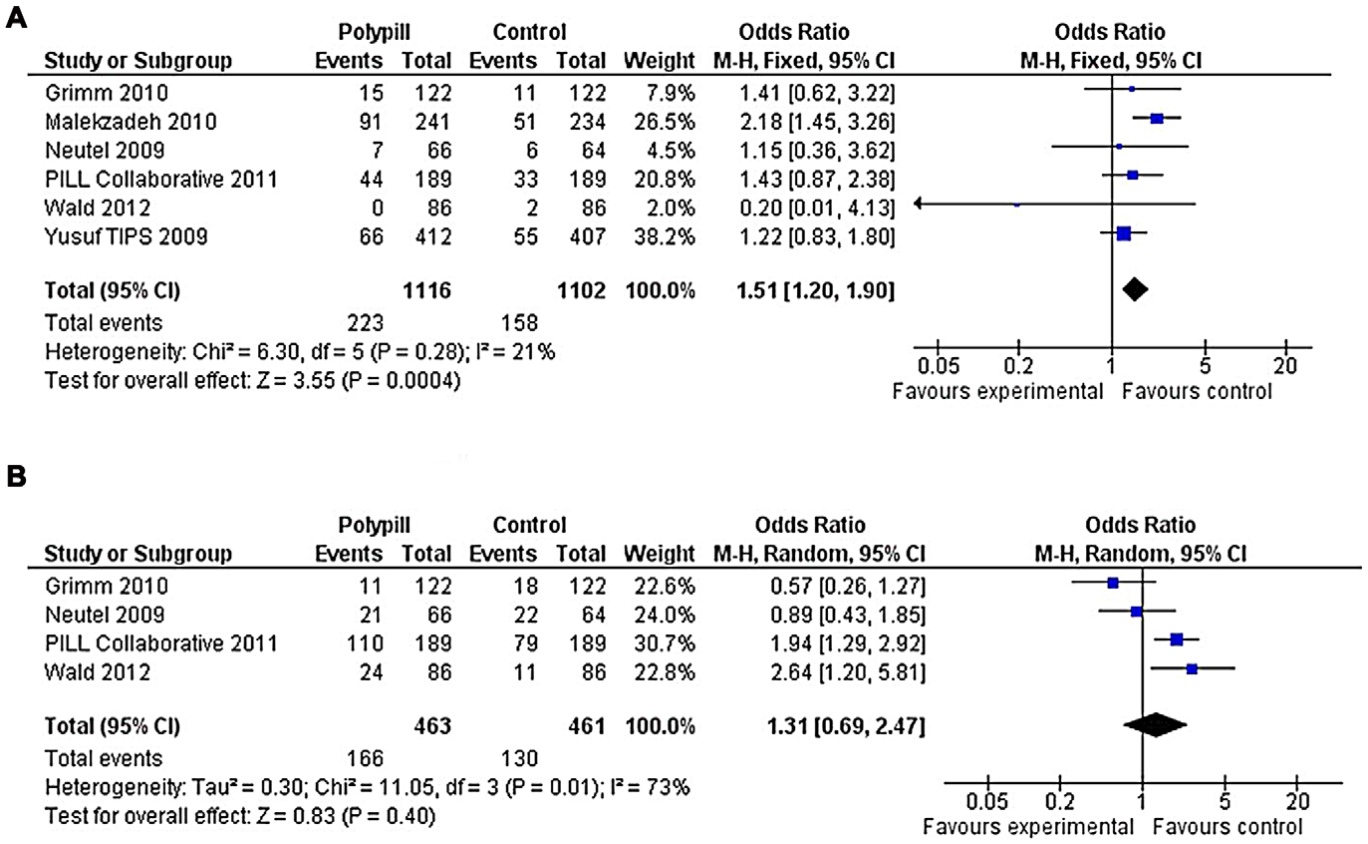
Forest Plots of Polypills versus Control for Change in Discontinuation of Study Medication and Side Effects.

Those taking ‘polypills’ were more likely to discontinue medication compared with placebo or one component (20% vs 14%) (OR: 1.5 (95%CI: 1.2, 1.9); Figure 4). There was lower heterogeneity (I^2^ = 21%) than for the estimates of effects on blood pressure or lipids. When only comparisons with placebo were included, [32], [33], [34] the odds ratio was 1.7 (95%CI: 1.3, 2.3) (24% vs 16%). Amongst the four trials that reported overall side effects [31], [32], [33], [36], the difference between ‘polypills’ and comparison arms in the proportion experiencing side effects (36% vs 28%) was not statistically significant (OR: 1.3 (95%CI: 0.7, 2.5; I^2^ = 73%) (Figure 4). The difference approached significance when only placebo-controlled trials were compared (45% vs 33%) (OR: 1.7 (95%CI: 0.97, 2.9)).

### Study Quality and Potential Bias

Due to the high levels of heterogeneity, quality-effects models were also conducted and compared with the results from random-effects models, using MetaXL in Excel. [27] Very similar effect estimates were obtained (Figure S2). Overall, included trials were of high quality (Jadad score 4/5 to 5/5), (Table 1). However, there were differences in baseline systolic blood pressure between intervention and control groups in the trials of the Pill Collaborative Group and Malekzadeh et al. (4.0 and 5.5 mmHg, respectively). [33], [34] The latter trial had imbalances in several baseline characteristics suggesting inadequacy of randomisation. [34] It also had moderately high rates of attrition from both groups but more in the intervention group (31% vs 22%), representing another potential source of bias. Risk of bias in this trial was therefore “uncertain” according to Cochrane criteria (Table S3). [24] There was no evidence of publication bias in any of the analyses (as evaluated by Begg’s and Egger’s tests and graphical representation using funnel plots (Figure S3)). The PRISMA checklist can be found for this meta-analysis in Table S4.

## Discussion

### Summary of Findings

Compared with placebo, the ‘polypills’ reduced systolic blood pressure by −9.2 mmHg, diastolic blood pressure by −5.0 mmHg, total cholesterol by −1.22 mmol/L and LDL-cholesterol by −1.02 mmol/L. Those taking a ‘polypill’ were more likely to discontinue study medication than those taking one component or placebo, although reported adverse effects were not significantly different.

### Strengths and Limitations

There was significant clinical and statistical heterogeneity amongst the trials. It may be argued that these studies should not have been combined in a meta-analysis because they contained interventions and controls with different components, and duration of follow-up varied from 6 weeks to 12 months. However, the meta-analysis assesses the use of cardiovascular ‘polypills’ in a variety of settings and populations, typical of real life, where at least one antihypertensive and one lipid-lowering medication have been combined in a fixed dose combination. Therefore, heterogeneity would be expected. Random-effects and quality-effects models found very similar effect sizes. [27] There are also limitations with using summary level data rather than individual-level data in a meta-analysis.

### Compared with the Literature and Implications for Future Practice

This meta-analysis reviewed the current evidence for efficacy and tolerability of cardiovascular ‘polypills’. The ‘polypills’ reduced risk factors compared with placebo; although less than has been estimated previously. [11] Wald et al estimated that a cardiovascular ‘polypill’ could reduce LDL cholesterol by 1.8 mmol/L and blood pressure by 20/11 mmHg. Actual reductions in risk factors depend on baseline risk factor levels and the number and doses of medications contained within the polypills. Wald’s estimated reduction in LDL cholesterol used a baseline of 4.8 mmol/L. [5] A 2003 meta-analysis of statin trials provides expected reductions in LDL based on statin and dose [5], from which expected reductions in LDL can be estimated for each of the trials in this meta-analysis taking into account baseline LDL level (Table 2). The ‘polypills’ in the trials included within this meta-analysis contained between one and three anti-hypertensives with doses of a quarter to twice the standard dose equivalent for each of these components (Table 2). The observed reductions in systolic blood pressure and LDL-cholesterol for the ‘polypills’ were comparable to that expected for two of the trials (Neutel and Wald). Although the observed reduction in systolic blood pressure was comparable to that expected in the PILL collaborative trial, the observed reduction in LDL-cholesterol was only 64% of that expected. The observed reduction in LDL cholesterol in the Grimm trial was 89% of that expected. The observed reduction in systolic blood pressure and LDL-cholesterol were much less than expected in the Malekzadeh and TIPS trials. This discrepancy could be explained by the greater loss to follow up in these trials, which would dilute treatment effect when ‘intention to treat’ analyses are undertaken, a lower adherence rate than reported, concomitant treatment in the control groups or methodological issues. However, it may also be closer to the real change in risk factors likely if used in practice. The trial that found the greatest reductions in blood pressure and lipids was the trial that had few participants drop-out, good adherence, and no concomitant blood pressure and lipid-lowering medication. [36] Almost all the participants had been taking the component medications prior to the trial, so presumably would be those most likely to tolerate and adhere to a combination ‘polypill’. [36] For this type of patient, we can expect predicted results. The real test will be in comparing ‘polypills’ to current care.

**Table 2:** Actual vs Expected Reductions in Systolic Blood Pressure and LDL-cholesterol in Trials of ‘Polypills’.

Trial	Actual vs Expected Reductions in Systolic Blood Pressure (SBP)						Actual vs Expected Reductions in LDL-Cholesterol				
	*Antihypertensive*	*Standard dose equivalent * [*50*]	*Mean baseline SBP mm Hg* *	*Expected reduction in* *SBP mmHg* **	*Observed mean difference in* *SBP mmHg*	*Observed/expected*	*Statin*	*Mean baseline LDL in mmol/l*	*Expected reduction in* *LDL mmol/l ?*	*Observed control-adjusted reduction in LDL mmol/l*	*Observed/expected*
Malekzadeh, 2010	Enalapril 2.5 mg	0.25	130	10.2 #	2.4	24%	Atorvastatin 20 mg	2.99	1.29	0.45	35%
	Hydrochlorothiazide 12.5 mg	0.5									
Neutel, 2009	Amlodipine 5 mg	1	150	8.7	8.3	95%	Atorvastatin 20 mg	3.46	1.49	1.55	104%
PILL collaboration, 2011	Lisinopril 10 mg	1	130	10.2 ##	9.9	97%	Simvastatin 20 mg	3.65	1.17	0.75	64%
	Hydrochlorothiazide 12.5 mg	0.5									
Wald, 2012	Hydrochlorothiazide 12.5 mg	0.5	140	17.6	17.9	100%	Simvastatin 40 mg	3.70	1.37	1.4	102%
	Losartan 25 mg	0.5									
	Amlodipine 2.5 mg	0.5									
The Indian Polycap Study, 2009	Hydrochlorothiazide 12.5 mg	0.5	130	18.2β	7.4	41%	Simvastatin 20 mg	3.00	0.96	0.72	75%
	Atenolol 50 mg	1									
	Ramipril 5 mg	2									
Grimm, 2010	N/A	N/A	N/A	N/A	N/A	NA	Atorvastatin 20 mg	3.35	1.44	1.28	89%

* rounded to nearest 10 mm Hg;

** based on mean baseline SBP & standard dose equivalence (from Law 2009) [4];

∧ mean baseline LDL × percentage reduction in LDL cholesterol for the statin at that dose (from Law 2003) [5]

# estimate: two drugs at half dose therefore an overestimate of likely effect;

## estimate: two drugs at half dose therefore an underestimate of likely effect; 12.7 mmHg for two drugs at standard dose;

β estimate: three drugs at standard dose; 15.2 mmHg for three drugs at half standard dose.

A short 12-week effectiveness trial comparing a ‘polypill’ with current care has been completed, but showed no difference between groups in systolic blood pressure or total cholesterol. [38] Several longer trials comparing a ‘polypill’ with current care are well underway or soon to be published. [39], [40], [41], [42], [43] The doses and number of components used will obviously influence both effectiveness and tolerability. A recent trial showed that doubling the doses of five ‘polypill’ components resulted in further significant reductions in systolic blood pressure (2.8 mmHg), diastolic blood pressure (1.7 mmHg), total cholesterol (0.19 mmol/L) and LDL-cholesterol (0.17 mmol/L). [37].

Even if the effectiveness of the ‘polypill’ strategy is found only to be equivalent to current care, cost is likely to be reduced, making preventive therapies more affordable, particularly for low-income countries. [22] A large part of the burden of chronic disease, particularly cardiovascular disease, is now borne by low-income countries. In 2005, it was estimated that 35 million people would die from non-communicable chronic diseases around the world. [22] Cardiovascular disease was found to be the leading single cause of death and accounted for 20% of the total disability-adjusted years lost amongst people over 30 years of age globally. Furthermore, 80% of the burden of chronic disease occurs in people under the age of 70 years. [22] Rates of preventive therapy are lower in low- and middle-income countries than in high-income countries. [44] Patented medications have made many of the cardiovascular preventive medications prohibitively expensive for these countries, severely limiting access to medications, increasing levels of poverty [45] and causing impoverishment in households struggling to afford medication. [46] The use of affordable ‘polypills’ could help address this issue.

A recent review of the cost-effectiveness of interventions for primary prevention of cardiovascular disease found that one of the ‘best value for money’ interventions was a combination of low cost blood pressure lowering medications and a statin, aimed at those at increased CVD risk. [47] Modelled cost-effectiveness analyses of a ‘polypill’ have also been promising in middle and higher income countries. [48], [49] However, the actual clinical and economic potential of a polypill strategy will require the results of effectiveness trials that compare ‘polypills’ with current care and subsequent cost-utility analyses.

## Supporting Information

Figure S1 **‘Polypills’ meta-analysis flow diagram of a second literature search.**
(DOCX)

Figure S2 **Meta-analyses comparing ‘quality effects’ models with ‘random effects’ or Mantel Haenszel fixed effects models undertaken in Excel.**
(DOCX)

Figure S3 **Funnel plots to assess for publication bias.**
(DOCX)

Table S1 **Literature search terms and results, conducted by CRE.**
(DOCX)

Table S2 **Literature search terms and results, conducted by AKG.**
(DOCX)

Table S3 **Risk of bias of included studies, using the Cochrane collaboration criteria.**
(DOCX)

Table S4 **PRISMA checklist.**
(DOCX)
