# Supplementary figures and images for: The Efficacy and Tolerability of ‘Polypills’: Meta-Analysis of Randomised Controlled Trials
Source: PLoS One. 2012 Dec 19;7(12):e52145. doi: 10.1371/journal.pone.0052145 (PMC3526586; doi:10.1371/journal.pone.0052145)

Figure S1: Polypills meta-analysis flow diagram of a second literature search


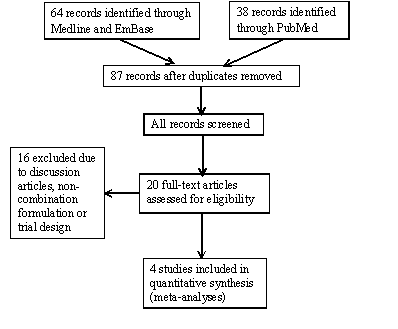

Supplement: Figure S1 — ‘Polypills’ meta-analysis flow diagram of a second literature search. (DOCX) [file pone.0052145.s001.docx]
