# Supplementary material for: The Efficacy and Tolerability of ‘Polypills’: Meta-Analysis of Randomised Controlled Trials
Source: PLoS One. 2012 Dec 19;7(12):e52145. doi: 10.1371/journal.pone.0052145 (PMC3526586; doi:10.1371/journal.pone.0052145)
Supplement: Figure S2 — Meta-analyses comparing ‘quality effects’ models with ‘random effects’ or Mantel Haenszel fixed effects models undertaken in Excel. (DOCX) [file pone.0052145.s002.docx]

Figure S2: Meta-analyses comparing ‘quality effects’ models with ‘random effects’ or Mantel Haenszel fixed effects models undertaken in Excel [[1](#_ENREF_1)]

1. Systolic blood pressure weighted mean difference (WMD) using quality effects (QE) and random effects (RE) models:


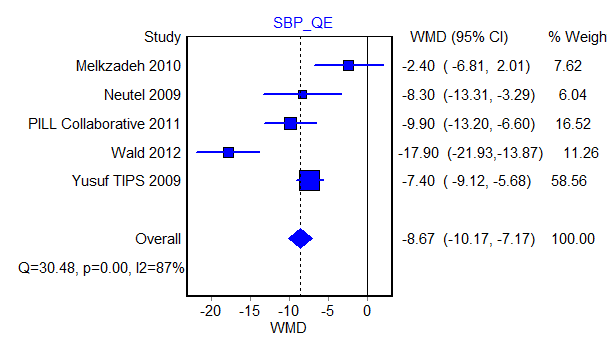


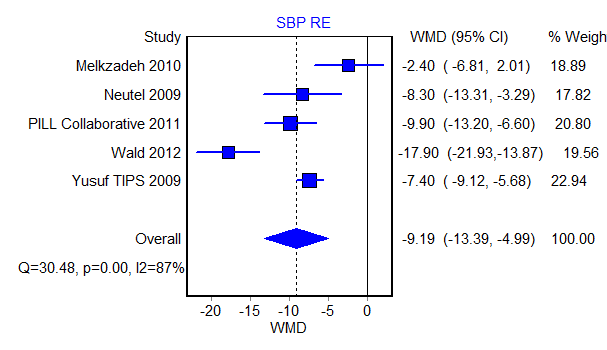


1. Diastolic blood pressure weighted mean difference (WMD) using quality effects (QE) and random effects (RE) models:


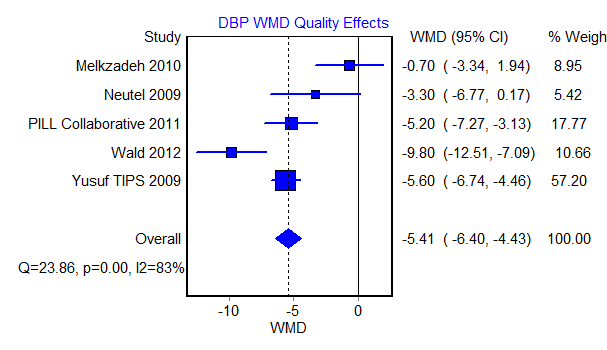


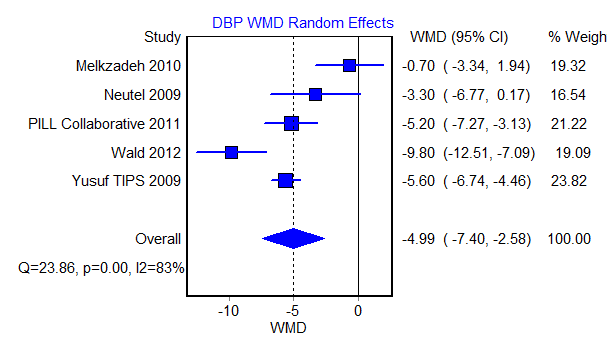


1. Total cholesterol weighted mean difference (WMD) using quality effects (QE) and random effects (RE) models:


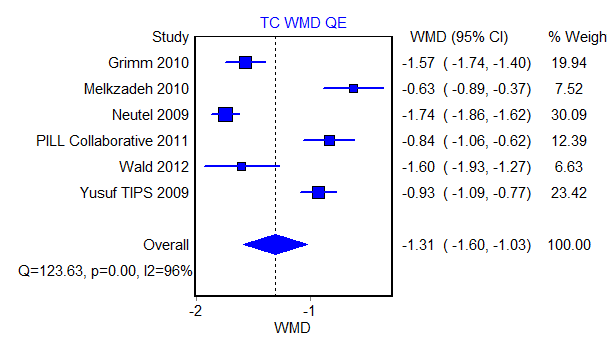


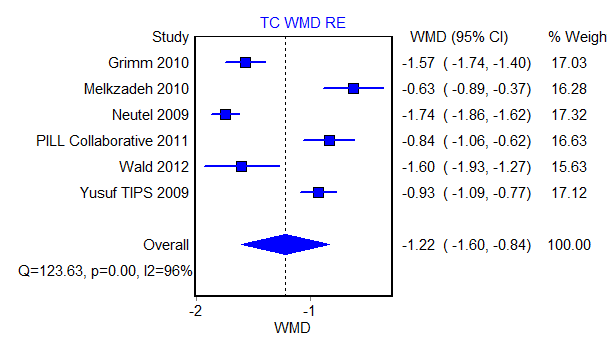


1. LDL-cholesterol weighted mean difference (WMD) using quality effects (QE) and random effects (RE) models:


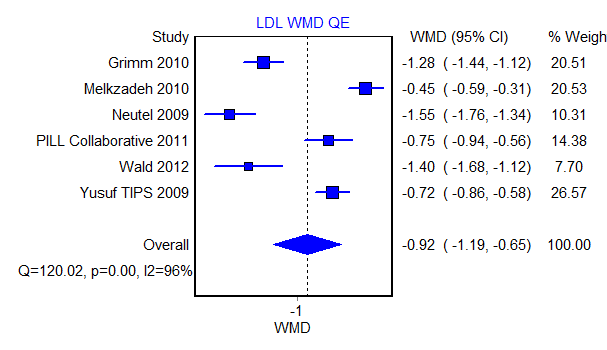


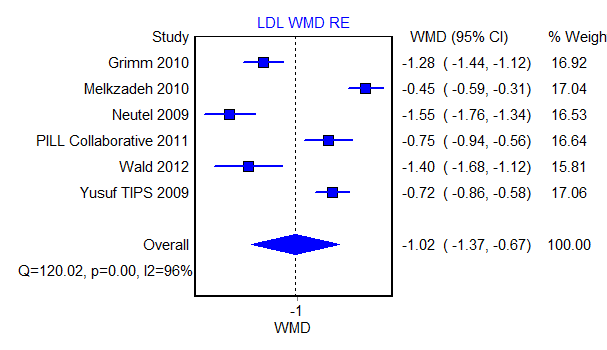


1. Discontinuation of study medication odds ratios (OR) using Quality effects (QE) and Mantel Haenszel fixed effects (MH) models:


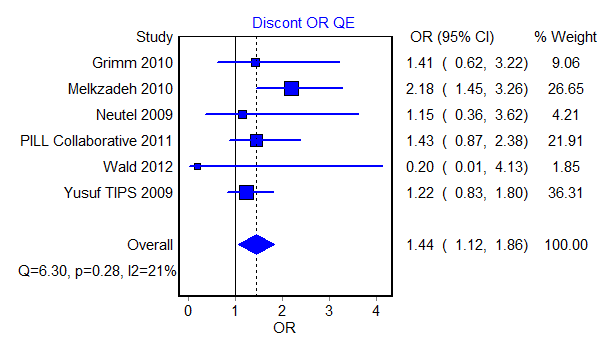


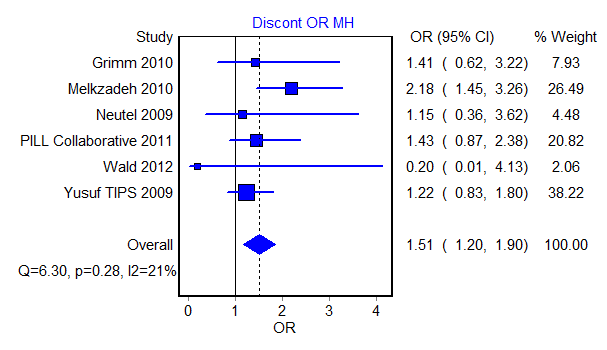


1. Side effects of study medication odds ratios (OR) using Quality effects (QE) and Random effects (RE) models:


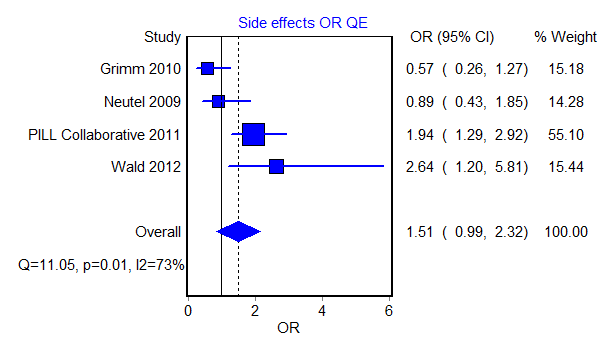


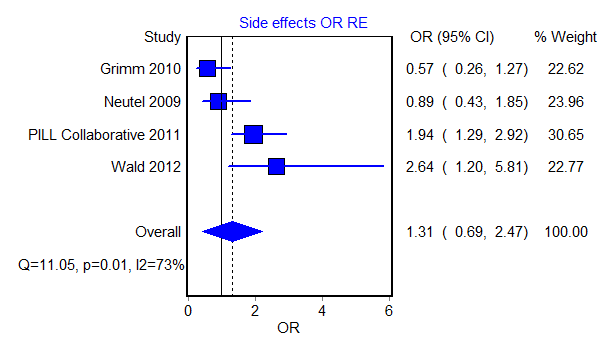


Reference:

1. Doi SAR, Barendregt JJ, Mozurkewich EL (2011) Meta-analysis of heterogeneous clinical trials: an empirical example. Contemporary Clinical Trials 32: 288-298.
