# Supplementary material for: The Efficacy and Tolerability of ‘Polypills’: Meta-Analysis of Randomised Controlled Trials
Source: PLoS One. 2012 Dec 19;7(12):e52145. doi: 10.1371/journal.pone.0052145 (PMC3526586; doi:10.1371/journal.pone.0052145)
Supplement: Figure S3 — Funnel plots to assess for publication bias. (DOCX) [file pone.0052145.s003.docx]

Figure S3: Funnel plots to assess for publication bias


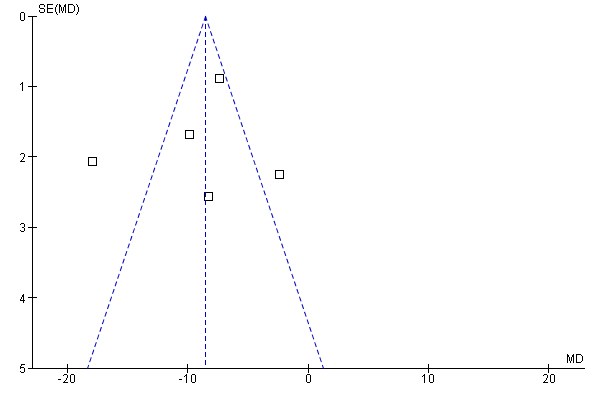


Systolic BP


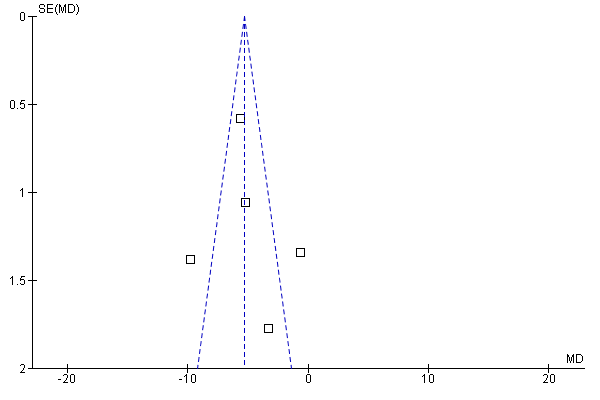


Diastolic BP


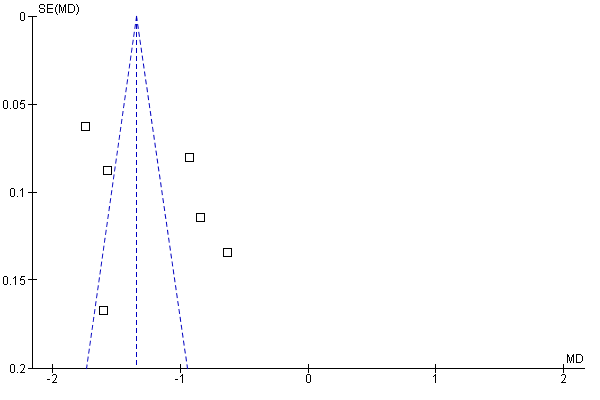


Total cholesterol


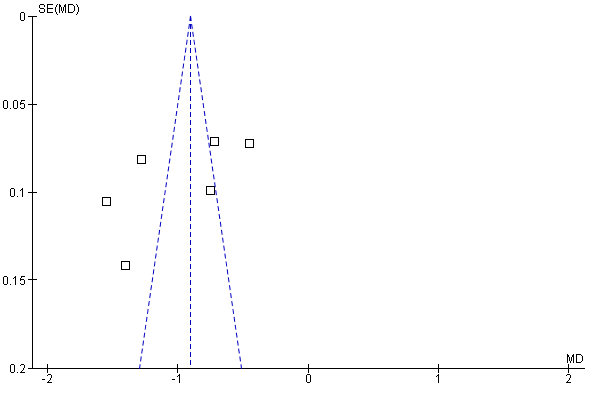


LDL-cholesterol


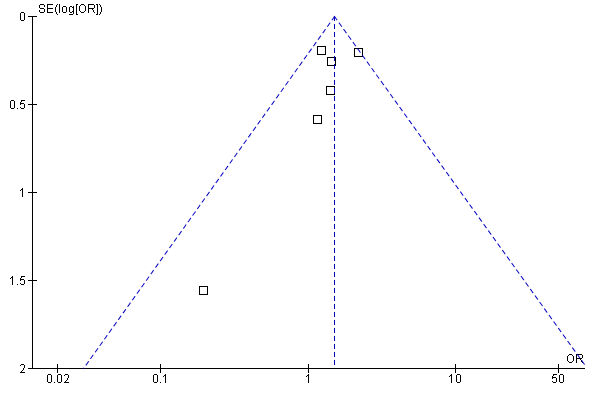


Discontinuation of trial medication


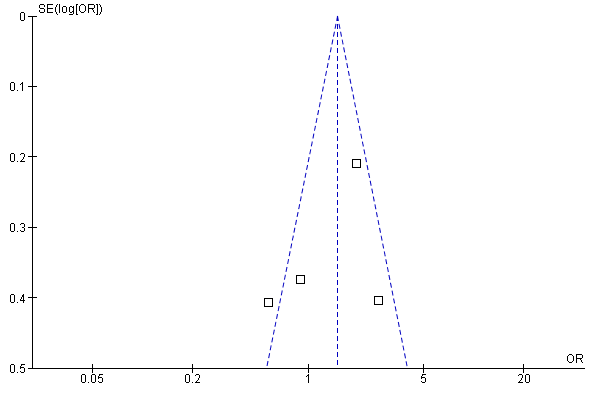


Side effects
