# Supplementary material for: The Efficacy and Tolerability of ‘Polypills’: Meta-Analysis of Randomised Controlled Trials
Source: PLoS One. 2012 Dec 19;7(12):e52145. doi: 10.1371/journal.pone.0052145 (PMC3526586; doi:10.1371/journal.pone.0052145)
Supplement: Table S1 — Literature search terms and results, conducted by CRE. (DOCX) [file pone.0052145.s004.docx]

Table S1: Literature search terms and results, conducted by CRE*

| ***Cochrane Central Register of Controlled Trials*** | | ***PubMed*** | | ***Medline*** | |
| --- | --- | --- | --- | --- | --- |
| 1. exp Antihypertensive Agents/ | 20324 | 1. Search randomized controlled trial [pt] | [322088](http://www.ncbi.nlm.nih.gov.ezproxy.auckland.ac.nz/pubmed/?cmd=HistorySearch&querykey=1) | 1. randomized controlled trial.pt. | 325488 |
| 1. exp Anticholesteremic Agents/ | 4131 | 1. Search controlled clinical trial [pt] | [83656](http://www.ncbi.nlm.nih.gov.ezproxy.auckland.ac.nz/pubmed/?cmd=HistorySearch&querykey=2) | 1. controlled clinical trial.pt. | 83975 |
| 1. 1 and 2 | 179 | 1. Search randomized [tiab] | [261246](http://www.ncbi.nlm.nih.gov.ezproxy.auckland.ac.nz/pubmed/?cmd=HistorySearch&querykey=3) | 1. randomized.ab. | 240956 |
| 1. polypill.mp. | 11 | 1. Search placebo [tiab] | [139959](http://www.ncbi.nlm.nih.gov.ezproxy.auckland.ac.nz/pubmed/?cmd=HistorySearch&querykey=4) | 1. placebo.ab. | 135287 |
| 1. 3 or 4 | 185 | 1. Search randomly [tiab] | [179730](http://www.ncbi.nlm.nih.gov.ezproxy.auckland.ac.nz/pubmed/?cmd=HistorySearch&querykey=5) | 1. clinical trials as topic.sh. | 159504 |
| 1. exp Drug Combinations/ | 8215 | 1. Search trial [tiab] | [301834](http://www.ncbi.nlm.nih.gov.ezproxy.auckland.ac.nz/pubmed/?cmd=HistorySearch&querykey=6) | 1. randomly.ab. | 177144 |
| 1. exp Cardiovascular Diseases/ | 58350 | 1. Search groups [tiab] | [1190802](http://www.ncbi.nlm.nih.gov.ezproxy.auckland.ac.nz/pubmed/?cmd=HistorySearch&querykey=7) | 1. trial.ti. | 103633 |
| 1. 5 and 6 and 7 | 14 | 1. Search #1 OR #2 OR #3 OR #4 OR #5 OR #6 OR #7 | [1752009](http://www.ncbi.nlm.nih.gov.ezproxy.auckland.ac.nz/pubmed/?cmd=HistorySearch&querykey=8) | 1. 1 or 2 or 3 or 4 or 5 or 6 or 7 | 780759 |
|  |  | 1. Search animals [mh] NOT humans [mh] | [3664037](http://www.ncbi.nlm.nih.gov.ezproxy.auckland.ac.nz/pubmed/?cmd=HistorySearch&querykey=9) | 1. exp animals/ not humans.sh. | 3703134 |
|  |  | 1. Search #8 NOT #9 | [1449710](http://www.ncbi.nlm.nih.gov.ezproxy.auckland.ac.nz/pubmed/?cmd=HistorySearch&querykey=10) | 1. 8 not 9 | 721957 |
|  |  | 1. Search Cardiovascular diseases [MeSH] OR myocardial infarction [MeSH] | [1703983](http://www.ncbi.nlm.nih.gov.ezproxy.auckland.ac.nz/pubmed/?cmd=HistorySearch&querykey=11) | 1. exp Antihypertensive Agents/ | 216584 |
|  |  | 1. Search Drug Combination[MeSH Terms] | [54315](http://www.ncbi.nlm.nih.gov.ezproxy.auckland.ac.nz/pubmed/?cmd=HistorySearch&querykey=12) | 1. exp Anticholesteremic Agents/ | 43770 |
|  |  | 1. Search Antihypertensive agents [MeSH] AND Anticholesterolemic agents [MeSH] OR Polypill [tiab] | [146](http://www.ncbi.nlm.nih.gov.ezproxy.auckland.ac.nz/pubmed/?cmd=HistorySearch&querykey=13) | 1. 11 and 12 | 1360 |
|  |  | 1. Search ((antihypertensive agents[MeSH Terms]) AND anticholesteremic agents[MeSH Terms]) OR polypill[Title/Abstract] | [839](http://www.ncbi.nlm.nih.gov.ezproxy.auckland.ac.nz/pubmed/?cmd=HistorySearch&querykey=14) | 1. polypill.mp. | 163 |
|  |  | 1. Search (((#10) AND #11) AND #12) AND #14 | [26](http://www.ncbi.nlm.nih.gov.ezproxy.auckland.ac.nz/pubmed/?cmd=HistorySearch&querykey=15) | 1. 13 or 14 | 1482 |
|  |  |  |  | 1. exp Drug Combinations/ | 54729 |
|  |  |  |  | 1. exp Cardiovascular Diseases/ | 1706336 |
|  |  |  |  | 1. 10 and 15 and 17 | 262 |
|  |  |  |  | 1. 18 and 16 | 32 |

*Conducted on 24^th^ April 2012
