# Supplementary material for: The Efficacy and Tolerability of ‘Polypills’: Meta-Analysis of Randomised Controlled Trials
Source: PLoS One. 2012 Dec 19;7(12):e52145. doi: 10.1371/journal.pone.0052145 (PMC3526586; doi:10.1371/journal.pone.0052145)
Supplement: Table S2 — Literature search terms and results, conducted by AKG. (DOCX) [file pone.0052145.s005.docx]

Table S2: Literature search terms and results, conducted by AKG

| *PubMed* | *Search Terms* | *Results* | *Medline* | *Search Terms* | *Results* |
| --- | --- | --- | --- | --- | --- |
| #1 | Search (((((randomized clinical trial) OR randomised clinical trial) OR randomized controlled trial) OR randomised controlled trial) OR randomized controlled trial[MeSH Major Topic]) OR randomised clinical trial[MeSH Major Topic] | 404035 | 1 | randomized clinical trial.af. | 25307 |
| #2 | Search (((randomly) OR randomised) OR randomized) OR random* | 763429 | 2 | randomized clinical trial.ab,cn,hw,kw,ot,pt,sh,sl,ti,tw | 25296 |
| #3 | Search ((((trial) OR clinical trial) OR study) OR trial[MeSH Major Topic]) OR trial[Title] | 5761319 | 3 | controlled clinical trial.pt. | 83971 |
| #4 | Search #2 OR #3 | 5909676 | 4 | controlled clinical trial.af. | 483908 |
| #5 | Search #2 AND #3 | 615072 | 5 | clinical trial.af. | 1489830 |
| #6 | Search #1 OR #5 | 615137 | 6 | (randomized clinical trial or randomised clinical trial).af. | 28430 |
| #7 | Search ((polypill) OR polycap) OR polypill[MeSH Subheading] | 150 | 7 | randomized controlled trial.af. | 677651 |
| #8 | Search (((drug combination) OR combination therapy) OR fixed combination) OR fixed dose combination | 556650 | 8 | (Trial or intervention or Study).af. | 15858322 |
| #9 | Search ((((antihypertensive) OR antihypertensive[MeSH Major Topic]) OR blood pressure medication) OR antihyperten*) OR blood pressure lowering medication | 260992 | 9 | random*.af. | 1613161 |
| #10 | Search (((drug) OR treatment) OR medication) OR agent | 9243118 | 10 | 8 and 9 | 1311829 |
| #11 | Search (((blood pressure) OR hypertension) OR hypertension[MeSH Major Topic]) OR blood pressure[MeSH Subheading] | 670035 | 11 | 1 or 2 or 3 or 4 or 5 or 6 or 7 or 10 | 2177666 |
| #12 | Search #11 and #10 | 417573 | 12 | (polypill or polycap).af. | 4293 |
| #13 | Search #12 AND #9 | 112866 | 13 | antihypertensive.af. | 161351 |
| #14 | Search ((((hyperlipdaemia) OR hyperlipidemia) OR hyperlipidaemia[MeSH Major Topic]) OR hypercholesterolemia) OR hypercholeterol* | 67707 | 14 | (Hypertension or high blood pressure).af. | 930375 |
| #15 | Search #14 and #10 | 42001 | 15 | (treatment or management).af. | 8671413 |
| #16 | Search (((((antihyperlipidaemic) OR statin) OR anticholestremic) OR lipid lowering agent) OR antihyperlipidemic[MeSH Major Topic]) OR anticholestermic[MeSH Major Topic] | 28461 | 16 | (drug or agent).af. | 8731102 |
| #17 | Search #16 AND #15 | 8065 | 17 | 14 and 15 and 16 | 143019 |
| #18 | Search #8 AND (#17 OR #15) AND (#16 OR #9) | 1831 | 18 | 13 or 17 | 256550 |
| #19 | Search #18 OR #7 | 1978 | 19 | (anticholesteremic or antihyperlipidaemic or Lipid lowering).af. | 32589 |
| #20 | Search (((((((cardiovascular disease) OR cardiovascular[MeSH Major Topic]) OR coronary artery disease) OR coronary heart disease) OR coronary heart disease[MeSH Major Topic]) OR stroke) OR cerebrovascular disease) AND cerebrovascular disease[MeSH Major Topic] | 198959 | 20 | (hyperlipidaemia or hyperlipidemia or high cholesterol).af. | 79664 |
| #21 | Search (((((((compliance) OR adherence) OR persistence) OR safety) OR blood pressure) OR complianc*) OR adverse events) OR cardiovascular outcome | 1037794 | 21 | 15 and 16 and 20 | 16123 |
| #22 | Search #21 OR #20 | 1213847 | 22 | 19 or 21 | 46500 |
| #23 | Search #22 AND #19 AND #6 | 336 | 23 | 18 and 22 | 8548 |
| #24 | Search (human) NOT animal | 10987182 | 24 | (drug combination or fixed cose combination or combination).af. | 1316016 |
| #25 | Search (English) OR English[Language] | 19078152 | 25 | 23 and 24 | 1301 |
| #26 | Search #23 AND #24 AND #25 | 323 | 26 | 12 or 25 | 5567 |
| #27 | Search #26 AND #7 | 16 | 27 | (cardiovascular disease or cardiovascular* or coronary*).af. | 2549447 |
|  |  |  | 28 | (cardiovascular disease or cardiovascular* or coronary*).af. | 2549447 |
|  |  |  | 29 | 11 and 26 and 28 | 523 |
|  |  |  | 30 | (Human or Men or women).af. | 17372214 |
|  |  |  | 31 | (Human or Men or women).af. | 17372214 |
|  |  |  | 32 | English.lg. or English.af. | 39431332 |
|  |  |  | 33 | 29 and 31 and 32 | 398 |
|  |  |  | 34 | blood pressure.lg. or systolic.af. or diastolic.af. | 349418 |
|  |  |  | 35 | cholesterol.lg. or LDL.af. or total cholesterol.af. or lipid.af. | 879637 |
|  |  |  | 36 | safety.lg. or adverse reaction.af. or adverse event.af. or advers*.af. | 2405028 |
|  |  |  | 37 | compliance.lg. or complian*.af. or adherence.af. or pill count.af. or mediation possession ratio.af. or persistence.af. | 525280 |
|  |  |  | 38 | 34 or 35 or 36 or 37 | 3967701 |
|  |  |  | 39 | 33 and 38 | 345 |
|  |  |  | 40 | remove duplicates from 39 | 334 |
|  |  |  | 41 | 12 and 40 | 64 |

NB: No further references were found when Embase was searched
