# Supplementary material for: The Efficacy and Tolerability of ‘Polypills’: Meta-Analysis of Randomised Controlled Trials
Source: PLoS One. 2012 Dec 19;7(12):e52145. doi: 10.1371/journal.pone.0052145 (PMC3526586; doi:10.1371/journal.pone.0052145)
Supplement: Table S3 — Risk of bias of included studies, using the Cochrane collaboration criteria. (DOCX) [file pone.0052145.s006.docx]

Table S3: Risk of bias of included studies, using the Cochrane collaboration criteria [[1](#_ENREF_1)]

| Study | Random sequence generation (selection bias) | Allocation concealment (selection bias) | Blinding of participants and personnel (performance bias) | Blinding of outcome assessment (detection bias) | Incomplete outcome data (attrition bias) Self-reported outcomes | Incomplete outcome data (attrition bias) Objective outcomes | Selective reporting (reporting bias) | Other bias |
| --- | --- | --- | --- | --- | --- | --- | --- | --- |
| Grimm | Low risk | Low risk | Low risk | Low risk | Low risk | Low risk | Low risk | Low risk |
| Malekzadeh | Unclear risk | Unclear risk | Unclear risk | Unclear risk | Unclear risk | Unclear risk | Unclear risk | Low risk |
| Neutel | Low risk | Low risk | Low risk | Low risk | Low risk | Low risk | Low risk | Low risk |
| Pill Collaboration | Low risk | Low risk | Low risk | Low risk | Low risk | Low risk | Low risk | Low risk |
| Wald | Low risk | Low risk | Low risk | Low risk | Low risk | Low risk | Low risk | Low risk |
| Yusuf TIPS 2009 | Low risk | Low risk | Low risk | Low risk | Low risk | Low risk | Low risk | Low risk |

**Characteristics and risk of bias of included studies:**

**Grimm 2010[**[**2**](#_ENREF_2)**]:**

| **Methods** | Double-blind RCT |
| --- | --- |
| **Participants** | “≥21 years of age, had hypertension, but no history of CVD or diabetes, and had ≥2 of the following CV risk factors: age ≥45 years if male; ≥55 years if female; current smoker; a family history of premature coronary heart disease (CHD) in a first-degree relative; high-density lipoprotein cholesterol (HDL-C) < 40 mg/dL (<1.0 mmol/L); or a waist circumference of 102 cm (≥40 inches) if male or 88 cm (≥35 inches) if female. All participants were previously treated with AML 5 mg, with blood pressure (BP) either controlled or stage 1 hypertension (systolic BP [SBP] ≤ 159 mm Hg and diastolic BP [DBP] ≤ 99 mm Hg) or controlled BP at 10 mg of amlodipine, in addition to a fasting low-density lipoprotein cholesterol (LDL-C) level at screening of ≥100 mg/dL and ≤170 mg/dL.”[[2](#_ENREF_2)] |
| **Interventions** | Participants were randomized to either amlodipine (AML) (5 to 10 mg) plus therapeutic lifestyle change (TLC) or amlodipine/atorvastatin (AML/ATO) single-pill therapy (5 to 10 mg/20 mg) plus TLC |
| **Outcomes** | Proportion attaining BP and LDL-cholesterol goals; Also recorded mean total cholesterol and mean LDL of each group (used in the meta-analysis) |

**Risk of bias table**

| **Bias** | **Authors' judgement** | **Support for judgement** |
| --- | --- | --- |
| Random sequence generation (selection bias) |  | “Double-blinding occurred using a central web/telephone computer-based tele-randomization system which dispensed two double-blind labelled bottles to participants at each dispensing visit”[[2](#_ENREF_2)] |
| Allocation concealment (selection bias) |  | see above |
| Blinding of participants and personnel (performance bias) |  | see above |
| Blinding of outcome assessment (detection bias) |  | see above |
| Incomplete outcome data (attrition bias) Self-reported outcomes |  | 12% (15/122) of intervention and 9% (11/122) of control were lost to follow-up at end. But outcomes used in meta-analysis were available on 96% (118/122) of intervention and 94% (115/122) of control participants. Also blinded. |
| Incomplete outcome data (attrition bias) Objective outcomes |  | 12% (15/122) of intervention and 9% (11/122) of control were lost to follow-up at end. But outcomes used in meta-analysis were available on 96% (118/122) of intervention and 94% (115/122) of control participants. Also blinded. |
| Selective reporting (reporting bias) |  |  |
| Other bias |  |  |

**Malekzadeh 2010[**[**3**](#_ENREF_3)**]:**

| **Methods** | Double-blind placebo-controlled trial (12 month after 8-week placebo run-in phase) |
| --- | --- |
| **Participants** | 475 participants, aged 50 to 79 years, without cardiovascular disease, hypertension or hyperlipidaemia |
| **Interventions** | Fixed-dose combination therapy with aspirin 81 mg, enalapril 2.5 mg, atorvastatin 20 mg and hydrochlorothiazide 12.5 mg (polypill) or placebo |
| **Outcomes** | LDL-cholesterol, systolic and diastolic blood pressure and adverse reactions over 12 months |

**Risk of bias table**

| **Bias** | **Authors' judgement** | | | **Support for judgement** | |
| --- | --- | --- | --- | --- | --- |
| Random sequence generation (selection bias) |  | | | Although randomisation was by "computer-generated list of numbers"[[3](#_ENREF_3)], there was imbalance of characteristics between intervention and control bringing into question the adequacy of randomisation | |
| Allocation concealment (selection bias) |  | | | Again, although randomisation was by "computer-generated list of numbers", and participants were "allocated the correspondingly numbered blister packs containing either the ‘polypill’ as a single tablet or an identical placebo"[[3](#_ENREF_3)], there was imbalance of characteristics between the groups bringing into question adequacy of allocation concealment. | |
| Blinding of participants and personnel (performance bias) |  | | | Although "Participants and researchers were blind to the allocation"[[3](#_ENREF_3)], attrition rates were different for the intervention and the control groups although this could have been due to difference in tolerability or clinical effect rather than ‘unblinding’. | |
| Blinding of outcome assessment (detection bias) |  | | | As above | |
| Incomplete outcome data (attrition bias) Self-reported outcomes |  | | | Attrition rates were high and different for the intervention and the control group over the duration of the trial, ie: 32% (76/241) in the intervention group and 22% (51/234) in the control group | |
| Incomplete outcome data (attrition bias) Objective outcomes |  | | | Attrition rates were high and different for the intervention and the control group over the duration of the trial, ie: 32% (76/241) in the intervention group and 22% (51/234) in the control group | |
| Selective reporting (reporting bias) |  | | | as above | |
| Other bias | |  |  | |  |
|  |  | | |  | |

**Neutel 2009[**[**4**](#_ENREF_4)**]:**

| **Methods** | 8-week double-blind placebo-controlled RCT |
| --- | --- |
| **Participants** | Men and women >21 years old and with hypertension (systolic blood pressure (SBP)>140-169 or diastolic BP (DBP)>90-105) and dyslipidaemia (LDL 2.85-4.14 mmol/L) but without previous CVD or diabetes and no treatment in previous 3 months. |
| **Interventions** | Amlodipine/atorvastatin (5/20mg) plus therapeutic lifestyle changes (TLC) vs placebo plus TLC. (After 4 weeks add-on anti-hypertensives and/or lipid lowering meds was permitted in both groups according to investigator). |
| **Outcomes** | Percentage of patients in whom both the BP and LDL treatment targets (BP <140⁄90 mm Hg7 and LDL <2.59 mmol ⁄L were reached at week 4 and week 8). Mean changes in BP and LDL at week 4 and week 8 were also available. Ten year Framingham risk score of coronary heart disease. |

**Risk of bias table**

| **Bias** | **Authors' judgement** | **Support for judgement** |
| --- | --- | --- |
| Random sequence generation (selection bias) |  | “Patients were randomized in a double-blind manner” [[4](#_ENREF_4)] to receive either both amlodipine⁄ atorvastatin (5 ⁄20 mg) and TLC or placebo and TLC. Balanced at baseline. |
| Allocation concealment (selection bias) |  | “Patients were randomized in a double-blind manner” [[4](#_ENREF_4)] to receive either both amlodipine⁄ atorvastatin (5 ⁄20 mg) and TLC or placebo and TLC. Balanced at baseline |
| Blinding of participants and personnel (performance bias) |  | Similar attrition rates. Blinded using placebo. |
| Blinding of outcome assessment (detection bias) |  |  |
| Incomplete outcome data (attrition bias) Self-reported outcomes |  |  |
| Incomplete outcome data (attrition bias) Objective outcomes |  |  |
| Selective reporting (reporting bias) |  |  |
| Other bias |  |  |

**PILL Collaborative 2011[**[**5**](#_ENREF_5)**]:**

| **Methods** | Double-blind placebo-controlled RCT with 12 weeks follow-up |
| --- | --- |
| **Participants** | Adults with 5-year cardiovascular risk of >7.5% but <15%. Concomitant medications were allowed at clinicians’ discretion during the trial if they became indicated. |
| **Interventions** | A ‘polypill’ (containing aspirin 75 mg, lisinopril 10 mg, hydrochlorothiazide 12.5 mg and simvastatin 20 mg) vs placebo |
| **Outcomes** | Systolic blood pressure, LDL and tolerability |

**Risk of bias table**

| **Bias** | **Authors' judgement** | **Support for judgement** |
| --- | --- | --- |
| Random sequence generation (selection bias) |  | Study treatments were allocated using a central computer-based randomisation service |
| Allocation concealment (selection bias) |  | Participants, research staff and and co-ordinating centre staff were all blinded to the allocation. |
| Blinding of participants and personnel (performance bias) |  | Use of identical placebo |
| Blinding of outcome assessment (detection bias) |  |  |
| Incomplete outcome data (attrition bias) Self-reported outcomes |  | High rates of follow-up (98% in intervention and 99% in the control) |
| Incomplete outcome data (attrition bias) Objective outcomes |  | High rates of follow-up (98% in intervention and 99% in the control) |
| Selective reporting (reporting bias) |  |  |
| Other bias |  |  |

**Wald 2012[**[**6**](#_ENREF_6)**]:**

| **Methods** | Randomized double-blind placebo-controlled crossover trial of 12 weeks duration (and comparison with predicted) |
| --- | --- |
| **Participants** | At least 50 years of age without a history of cardiovascular disease (almost all had been on all the polypill components prior to starting) |
| **Interventions** | A ‘polypill’ (amlodipine 2.5 mg, losartan 25 mg, hydrochlorothiazide 12.5 mg and simvastatin 40 mg) each evening for 12 weeks and a placebo each evening for 12 weeks in random sequence |
| **Outcomes** | Change in systolic and diastolic blood pressure, LDL and total cholesterol (non-fasting) over 12 weeks compared with placebo |

**Risk of bias table**

| **Bias** | **Authors' judgement** | **Support for judgement** |
| --- | --- | --- |
| Random sequence generation (selection bias) |  | The randomization sequence was generated in advance by a computer random number generator, in blocks of 4. |
| Allocation concealment (selection bias) |  | Neither the participants nor the investigators knew the sequence (ie: double-blind). |
| Blinding of participants and personnel (performance bias) |  | Placebo-controlled (double-blind) |
| Blinding of outcome assessment (detection bias) |  |  |
| Incomplete outcome data (attrition bias) Self-reported outcomes |  | 98% follow-up rate |
| Incomplete outcome data (attrition bias) Objective outcomes |  | 98% follow-up rate |
| Selective reporting (reporting bias) |  |  |
| Other bias |  |  |

**Yusuf TIPS 2009[**[**7**](#_ENREF_7)**]:**

| **Methods** | Double-blind randomised controlled trial, multi-centred, 9-armed |
| --- | --- |
| **Participants** | “2053 individuals without cardiovascular disease, aged 45–80 years and with one risk factor”… “(type 2 diabetes; blood pressure >140 mm Hg systolic or 90 mm Hg diastolic, but <160/100 mm Hg; smoker within past 5 years; increased waist to hip ratio [>0·85 for women and >0·90 for men]; or abnormal lipids [LDL cholesterol >3·1 mmol/L or HDL cholesterol <1·04 mmol/L])” [[7](#_ENREF_7)]. 18-24% of each group was also on a Ca channel blocker throughout the trial. |
| **Interventions** | The polycap (n=412) consisting of low doses of thiazide (12·5 mg), atenolol (50 mg), ramipril (5 mg), simvastatin (20 mg), and aspirin (100 mg) per day, or to eight other groups, each with approximately 200 individuals, of aspirin alone, simvastatin alone, hydrochlorothiazide alone, three combinations of the two blood-pressure-lowering drugs, three blood-pressure-lowering drugs alone, or three blood-pressure-lowering drugs plus aspirin. |
| **Outcomes** | LDL-cholesterol, blood pressure, heart rate; urinary 11-dehydrothromboxane B2; rates of discontinuation of drugs over 12 weeks (or last assessment). Outcomes assessed at baseline, 10 days, 4, 8, 12 and 16 weeks. Study drug was discontinued at 12 weeks. Outcome assessment 12 weeks or latest assessment prior with repeated measures modelling strategy to analyse outcomes recorded at four time-points after randomisation. |
| **Notes** | No placebo arm. Postulated Polycap would be non-inferior to combination of three drugs to lower blood pressure alone or in conjunction with aspirin. If non-inferiority was confirmed, then all groups with three blood-pressure- lowering drugs would be compared with groups with two drugs, with one drug, and with no blood-pressure-lowering drug to assess the incremental effects of addition of drugs. Funded by Cadila Pharmaceuticals, Ahmedabad, India (on steering committee but no role in data collection analysis, interpretation or write-up). |

**Risk of bias table**

| **Bias** | **Authors' judgement** | **Support for judgement** |
| --- | --- | --- |
| Random sequence generation (selection bias) |  | Randomisation by central secure website |
| Allocation concealment (selection bias) |  | Baseline assessments carried out prior to randomisation at distant site |
| Blinding of participants and personnel (performance bias) |  | Double-blind. All participants received single capsule |
| Blinding of outcome assessment (detection bias) |  | Double-blind assessments. Electronic sphygmomanometers used according to a protocol and fasting lipids in standard laboratory. |
| Incomplete outcome data (attrition bias) Self-reported outcomes |  | Participants were blind to capsule contents when reporting side effects or reason for discontinuation. |
| Incomplete outcome data (attrition bias) Objective outcomes |  | Final scheduled follow-up not available in 326 individuals. (Varied from 9-19% in different groups). However, at least one recording of blood pressure after randomisation was available in 1971 patients (96% - varied from 93-99% between groups). Obtained fasting blood samples at last scheduled visit on treatment for 1665 (81%) patients. 1874 (91%- varied from 90-95%) patients had at least one blood sample available after randomisation for analysis of lipids. (Also, some had 8 week follow-up due to expiry of polycap in Sep 2008.) |
| Selective reporting (reporting bias) |  |  |
| Other bias |  | As some components have predictive effects (e.g. Atenolol reduces pulse, simvastatin reduces lipids, and all BP lowering components lower BP, then there would be some risk of guessing allocation but this bias was minimised in objective measures but may have affected choice of discontinuation.) |

References:

1. Cochrane Collaboration (2011) Cochrane Handbook for Systematic Reviews of Interventions, Version 5.1.0. In: Higgins J, Green S, editors.

2. Grimm R, Malik M, Yunis C, Sutradhar S, Kursun A, et al. (2010) Simultaneous treatment to attain blood pressure and lipid goals and reduced CV risk burden using amlodipine/atorvastatin single-pill therapy in treated hypertensive participants in a randomized controlled trial. Vascular health and risk management 6: 261-271.

3. Malekzadeh F, Marshall T, Pourshams A, Gharravi M, Aslani A, et al. (2010) A pilot double-blind randomised placebo-controlled trial of the effects of fixed-dose combination therapy ('polypill') on cardiovascular risk factors. Int J Clin Pract 64: 1220-1227.

4. Neutel JM, Bestermann WH, Dyess EM, Graff A, Kursun A, et al. (2009) The use of a single-pill calcium channel blocker/statin combination in the management of hypertension and dyslipidemia: a randomized, placebo-controlled, multicenter study. Journal of clinical hypertension (Greenwich, Conn) 11: 22-30.

5. Pill Collaborative Group, Rodgers A, Patel A, Berwanger O, Bots M, et al. (2011) An international randomised placebo-controlled trial of a four-component combination pill ("polypill") in people with raised cardiovascular risk. PLoS ONE 6: e19857.

6. Wald DS, Morris JK, Wald NJ (2012) Randomized polypill crossover trial in people aged 50 and over. PLoS ONE 7: e41297.

7. The Indian Polycap Study (TIPS) (2009) Effects of a polypill (Polycap) on risk factors in middle-aged individuals wihtout cardiovascular disease (TIPS): a phase II, double-blind, randomised trial Lancet 373: 1341-1351.
